# A case report of esophageal leiomyoma in Alport's syndrome treated with robotic-assisted distal myotomy: A surgical technique to avoid esophagectomy^☆^

**DOI:** 10.1016/j.ijscr.2023.108433

**Published:** 2023-06-21

**Authors:** Flavio Roberto Takeda, Jose Donizeti de Meira Junior, Rubens Antonio Aissar Sallum

**Affiliations:** Department of Gastroenterology, Digestive Surgery Division, Hospital das Clínicas, University of São Paulo Medical School, Brazil

**Keywords:** Esophagectomy, Leiomyomatosis, Case report, Robotic surgical procedures, Dysphagia

## Abstract

**Introduction:**

Alport's syndrome is the most common hereditary nephropathy, characterized by progressive renal failure, sensorineural deafness, and ocular abnormalities. It may rarely coexist with diffuse leiomyomatosis of the digestive tract, respiratory tract, or female genitalia, and in this setting, it is called Alport-leiomyomatosis syndrome. The leiomyomas most commonly affect the esophagus, and the symptoms have early onset. Treatment is usually esophagectomy.

**Case presentation:**

We report the case of an 8 years-old girl in which we performed a novel strategy of an esophagus-sparing approach with a robotic-assisted myotomy. This conservative approach has never been described in the literature to our knowledge.

**Discussion:**

The underpinning rationale was to resolve the patient's symptoms with partial resection of the benign tumor, avoiding an esophagectomy. Although it is likely related to a higher relapsing rate, it is more tolerable by an 8 years-old patient, and was highly effective in resolving her symptoms.

**Conclusion:**

The video of a successful minimally invasive conservative approach to esophageal leiomyomatosis is presented.

## Highlights

• Alport's syndrome is the most common hereditary nephropathy.
• It may coexist with diffuse leiomyomatosis.
• Leiomyomas affect most commonly the esophagus.
• Treatment is usually esophagectomy.
• We present a novel minimally invasive conservative approach.

## 1 Introduction

Alport's syndrome is the most common hereditary nephropathy, characterized by progressive renal failure, sensorineural deafness, and ocular abnormalities [1]. It has a dominant X-linked inheritance, with an incidence of 1 per 40,000 to 50,000 people [2,3]. It may rarely coexist with diffuse leiomyomatosis of the digestive tract, respiratory tract, or female genitalia in approximately 2 to 5 % of Alport syndrome patients with a chromosomal microdeletion in the COL4A5 gene [4,5]. In this setting, it is called Alport-leiomyomatosis syndrome [6], described in 1983 [7]. The leiomyomas most commonly affect the esophagus, involving all its muscle layers, and the symptoms have early onset [4]. Patients suffer from dysphagia, odynophagia, retrosternal pain, and regurgitation. Treatment is usually the partial or total resection of the affected esophagus [6,8]. The following case, reported in line with the SCARE criteria [9], illustrates a new approach to treating this symptomatic leiomyomatosis.

## 2 Case report

We report the case of an 8 years-old girl previously diagnosed with gastroesophageal reflux disease, with a medical record of 3 episodes of aspiration pneumonia. Her complaints were retrosternal pain and dysphagia. She also had some hematuria episodes in the past. Her physical examination was unremarkable. Barium radiography evidenced esophageal dilation and impaired esophageal clearance. A presumptive diagnosis of achalasia was made, and a peroral endoscopic myotomy (POEM) was performed. An esophageal nodule was biopsied during the procedure, and pathology reported a leiomyoma. The patient's symptoms relapsed one month after POEM. The patient was then referred to a tertiary center. Thoracic magnetic resonance imaging revealed diffuse esophageal leiomyomatosis. Alport's syndrome diagnosis was confirmed after genetic evaluation. There were no ophthalmic or hearing impairments, and a nephrology assessment was initiated. Surgical treatment for esophageal leiomyomatosis was proposed. Due to the patient's young age, we programmed a robotic-assisted conservative procedure to preserve the esophagus. However, we obtained consent for an esophagectomy, if needed.

A senior upper GI surgeon performed the procedure. Initially, we performed an esophagogastric transition dissection with a total exposure of the distal esophagus. Once the distal esophagus was dissected and repaired, we started a myotomy until mucosa/submucosa was clearly visible. Then, we dissected the submucosal plane from the muscle layer containing the leiomyoma and removed a stripe of leiomyoma, leaving the mucosa exposed. Hiatoplasty and a partial posterior fundoplication were performed.

**Fig. 1:**
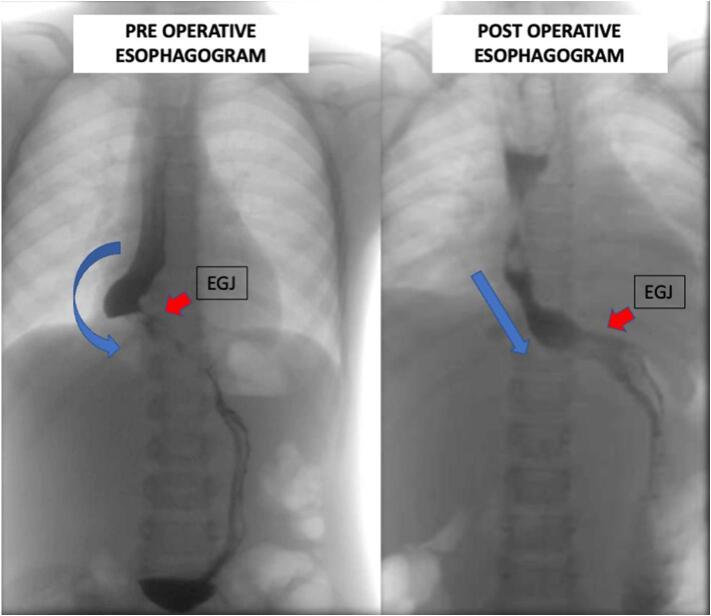
Comparison between preoperative (left) and postoperative (right) esophagogram, showing a significantly better esophageal clearance and adequacy of the esophageal axis and esophagogastric junction (EGJ) positioning.

The postoperative period was uneventful, and the patient was discharged on the fifth postoperative day with liquid oral intake. Barium radiography was performed on the 30th postoperative day and revealed a significantly better esophageal clearance than the preoperative exam (Fig. 1). The patient now tolerates a full diet without dysphagia or retrosternal pain complaints.

Written informed consent was obtained from the patient's parents to publish this case report and accompanying images. A copy of the written consent is available for review by the Editor-in-Chief of this journal on request.

Ethical approval for this study (Ethical Committee N° CCEP 1688/20) was provided by the Ethical Committee of our institution, on May 13, 2020.

## 3 Discussion

The conservative approach attempted, in this case, has never been described in the literature to our knowledge. The underpinning rationale was to resolve the patient's symptoms with partial resection of the benign tumor, avoiding an esophagectomy, which carries a risk of any postoperative adverse event of 63.9 % [10], anastomotic leak of 11.4 % [11], and death of 3.3 % [10]. This conservative approach is more tolerable although it is probably associated with a higher relapsing rate. It carries a more acceptable risk for an 8 years-old patient with a benign disease. It was highly effective in resolving her symptoms, as illustrated by the diet tolerance and the postoperative barium radiography.

## 4 Conclusion

We present a novel video of a minimally invasive conservative approach to esophageal leiomyomatosis, successfully relieving patients' symptoms without complications.

The following is the supplementary data related to this article.

Video 1 Esophageal leiomyomatosis low. Video 1

## Ethical approval

Case reports are exempt of ethical approval in our institution (Sao Paulo University) by the Ethics Committee of Sao Paulo University with the number 279.089 (online registry 10587).

## Funding

There was no financial support for this study.

## Author contribution

FRT: Conceptualization, review, formal analysis.

JDMJ: writing – writing and editing.

RAAS: writing and Supervision.

## Guarantor

FRT.

## Research registration number

This is not a first in man study.

## Declaration of competing interest

Authors declare that there is no actual or potential conflict of interest in relation to this article.
